# Epigenomic Interactions Between Chronic Pain and Recurrent Pressure Injuries After Spinal Cord Injury

**DOI:** 10.3390/epigenomes9030026

**Published:** 2025-07-23

**Authors:** Letitia Y. Graves, Melissa R. Alcorn, E. Ricky Chan, Katelyn Schwartz, M. Kristi Henzel, Marinella Galea, Anna M. Toth, Christine M. Olney, Kath M. Bogie

**Affiliations:** 1 VA Northeast Ohio Healthcare System, Cleveland, OH 44106, USA; mxa1176@case.edu (M.R.A.); mary.henzel@va.gov (M.K.H.); kmb3@case.edu (K.M.B.); 2 School of Nursing, University Texas Medical Branch at Galveston, Galveston, TX 77555, USA; 3 Department of Population and Quantitative Health Sciences, Case Western Reserve University, Cleveland, OH 44106, USA; erc6@case.edu; 4 James J Peter VA Medical Center, Bronx, NY 10468, USA; 5 Minneapolis VA Health Care System, Minneapolis, MN 55417, USA

**Keywords:** spinal cord injury, secondary health condition, neuropathic pain, chronic pain, recurrent pressure injury, DNA methylation

## Abstract

Background/Objectives: This study investigated variations in DNA methylation patterns associated with chronic pain and propensity for recurrent pressure injuries (PrI) in persons with spinal cord injury (SCI). Methods: Whole blood was collected from 81 individuals with SCI. DNA methylation was quantified using Illumina genome-wide arrays (EPIC and EPICv2). Comprehensive clinical profiles collected included secondary health complications, in particular current PrI and chronic pain. Relationships between recurrent PrI and chronic pain and whether the co-occurrence of both traits was mediated by changes in DNA methylation were investigated using R packages limma, DMRcate and mCSEA. Results: Three differentially methylated positions (DMPs) (cg09867095, cg26559694, cg24890286) and one region in the micro-imprinted locus for *BLCAP/NNAT* are associated with chronic pain in persons with SCI. The study cohort was stratified by PrI status to identify any sites associated with chronic pain and while the same three sites and region were replicated in the group with no recurrent PrI, two novel, hypermethylated (cg21756558, cg26217441) sites and one region in the protein-coding gene *FDFT1* were identified in the group with recurrent PrI. Gene enrichment and genes associated with specific promoters using MetaScape identified several shared disorders and ontology terms between independent phenotypes of pain and recurrent PrI and interactive sub-groups. Conclusions: DMR analysis using mCSEA identified several shared genes, promoter-associated regions and CGI associated with overall pain and PrI history, as well as sub-groups based on recurrent PrI history. These findings suggest that a much larger gene regulatory network is associated with each phenotype. These findings require further validation.

## 1. Introduction

Although symptoms are frequently studied individually, they seldom occur in isolation. Symptom science research has focused on quantifying subjective symptom experiences and measuring the physiologic and biologic or “omic” underpinnings of symptoms and sequelae common to health conditions and their treatments. Symptoms experienced from a central disease process or injury, such as SCI, are more likely to be multitudinous and co-occurring. Hunter-Revell demonstrated that chronic pain, depression, and spasticity, were often co-occurring or “clustered” symptoms which subsequently interfered with quality of life and functional activity, as well as psychological distress in individuals following SCI [1]. Although progress has been made in understanding the symptom experience in other significant diseases, the lack of symptom science research in the SCI population has hampered development of evidence-based interventions to improve outcomes for these individuals.

Precision health takes into account differences in an individual’s genes, environment, and lifestyle to identify risk(s), plan personalized treatment and devise prevention strategies. There continues to be much debate about the benefits of precision approaches in general, and even more so in rehabilitation, where individual heterogeneity is confounded by injury characteristics and treatment response. However, rehabilitation is the ideal specialty to employ epigenomics, as physiatrists seek to reduce disability in individuals with health conditions in interaction with their environment, thus bringing together a comprehensive multidisciplinary team to address complex medical management. Studies have demonstrated key relationships among the genome, environment, prenatal exposure to disease risk and prevention of secondary chronic co-morbidities [2,3]. Several studies have used in vitro and SCI preclinical models to study spinal cord circuits involved in neuropathic pain and epigenetic regulation [4,5,6], but to our knowledge, there have been no epigenome-wide studies conducted on the experience of chronic pain, specifically for individuals with SCI.

Even less is understood about epigenetic regulation of pressure injury (PrI) development, a common secondary health complication for persons with SCI or subsequent epigenetic signals influencing healing of chronic PrI. While there are modest numbers of genome-wide association studies (GWAS), no epigenetic-wide association (EWAS) studies were identified for PrI development. Epigenomic mechanisms of regulation and their relationship with SHC offer opportunities to inform SCI specific SHCs and co-morbidity, but the literature remains underdeveloped [7].

This study’s aim was to investigate if there is significant variation in genome-wide DNA methylation patterns in persons with SCI associated with experience of chronic pain and propensity for recurrent PrI. Changes in genome-wide DNA methylation (ΔDNAme) associated with recurrent PrI and experience of chronic pain were identified using two different models:

(1) ΔDNAme~PrI + Pain + PrI*Pain
(2) ΔDNAme~Pain|PrI

The aim of the first model is to identify changes in DNA methylation associated with both phenotypes, PrI and Pain in the entire cohort, whereas the aim of model 2 is to perform sub-group analysis and identify any shared, differentially methylated sites or regions associated with chronic pain experience given PrI history. We have found that indeed there are several differentially methylated sites associated with chronic pain, which vary depending upon whether an individual experiences recurrent PrI, in a predominantly male cohort of Veterans with chronic SCI. Further work is needed to validate these findings with larger, independent cohorts of people with SCI.

## 2. Results

### 2.1. Study Overview and Characteristics

**Figure 1:**
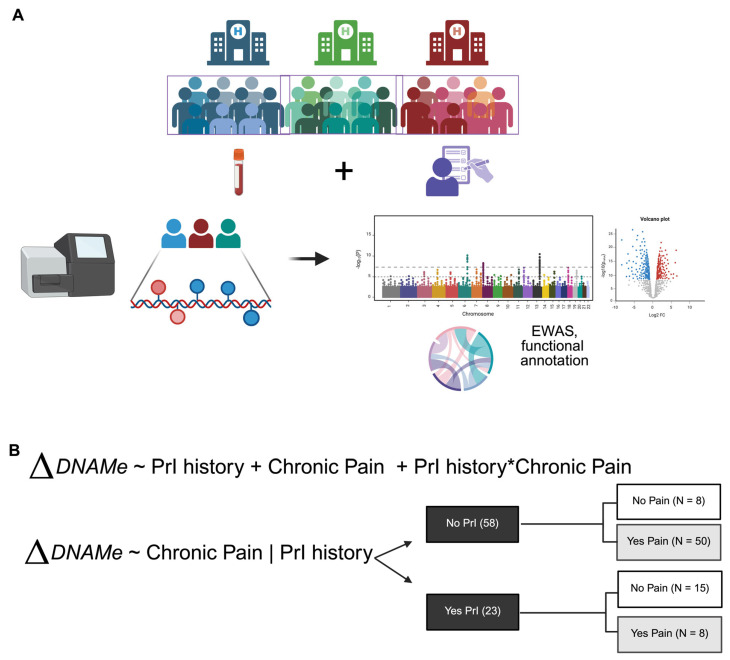
Overall study workflow. (**A**) Pipeline of sample collection through analysis. (**B**) Models chosen for dissection of epigenetic factors associated with secondary health complications, PrI and Pain, in Veterans with SCI at time of enrollment. Asterisk (*) indicates interaction between two phenotypes or terms in model 1. Changes in DNA methylation associated with phenotype groups are denoted as ΔDNAM. Created in BioRender. Alcorn, M. (2025) https://biorender.com/ji84z11 (accessed on 3 July 2025).

This observational study was conducted at three VA SCI Centers across the USA. Whole blood and clinical information were collected at time of enrollment to evaluate the incidence of SHCs related to SCI, including PrI history and chronic pain. Clinical information included the duration, grade and level of SCI. (Figure 1, Table 1).

### 2.2. EWAS Identifies Differentially Methylated Sites Associated with Chronic Pain in Persons with SCI (Model 1)

SCI is a highly heterogeneous condition with significant variation in neurological loss and other co-morbidities [8,9]. Based on our covariate and modeling analysis, we included the terms Pain, PrI, an interaction term and several support vectors. In order to develop a model that would increase our statistical power of identifying differentially methylated probes or sites (DMPs), we considered five different models accounting for different covariates identified by pair-wise correlation analysis (Kendall’s tau, adjusted *p*-values < 0.05), logistic regression, and biologically relevant variables known to influence DNA methylation levels such as age and history of smoking (Supplemental Tables S2 and S3, Supplemental Figure S1). We also tested versions of these models including support vectors calculated using the sva package in R to account for additional variation, such as any variability in immune-cell populations present in whole blood. To choose ideal models for DMP analysis, we calculated lambda GC values from summary statistics for each phenotype of chronic pain, recurrent PrI history and the interactive term [10] (Supplemental Table S4). Data was collected in batches and separate platforms (EPICv1 vs. EPICv2); therefore, we added sample platform as a covariate to all models, including those with support vectors (See Supplemental Figures S2 and S3 for principal component analysis before and after batch-correction using removeBatchEffect() function in limma).

**Figure 2:**
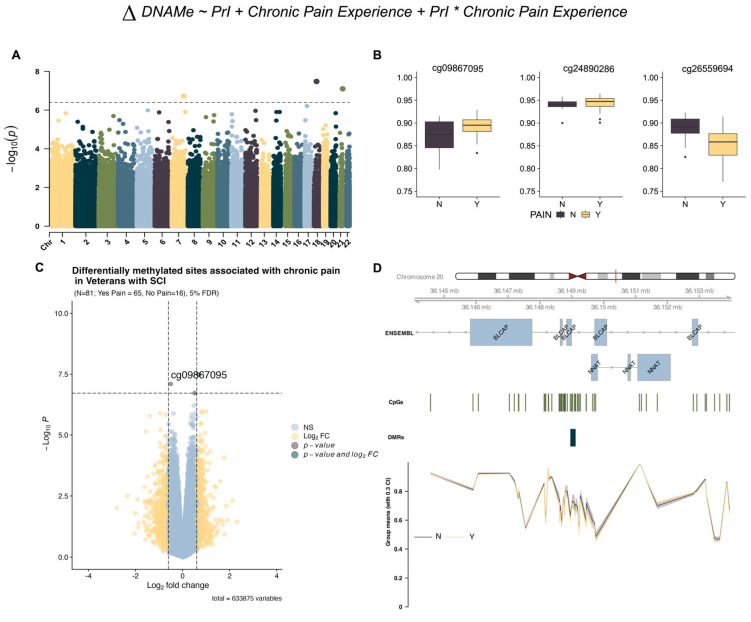
EWAS identifies genome-wide variation in DNA methylation associated with chronic pain. (**A**) Manhattan plot depicting −log10-transformed *p*-values per site in EPIC v1 (hg19) array for chronic pain using model 1. Asterisk indicates interaction term between both phenotypes (**B**) Individual effect plots of median and inter-quartile range (IQR) beta intensity for each significant differentially methylated probe indicate modest effect sizes from individual sites. (**C**) Volcano plot indicates that only one probe meets both the significance threshold and the log2fold-change threshold for genome-wide significance. (**D**) Differentially methylated region analysis using DMRcate and summary statistics identify one locus associated with chronic pain in imprinted locus containing protein coding genes *BLCAP* and *NNAT*. Grouped means with 30% confidence intervals for beta values are depicted in the lowest portion of the figure. The X-axis corresponds to genomic coordinates from ENSEMBL for locus containing the genes *BLCAP/NNAT*. Changes in DNA methylation associated with phenotype groups are denoted as ΔDNAMe. Created in BioRender. Alcorn, M. (2025) https://biorender.com/25f8dki (accessed on 3 July 2025).

Using limma and support vectors identified by R package sva (version 3.56.0), we identified three DMPs associated with chronic pain from model 1 (Table 2) but no sites associated with PrI or the interaction term (Figure 1B and Figure 2A). Effect plots of individual sites (Figure 2B) and the volcano plot (Figure 2C) indicate that each probe has a modest log2-fold change in beta intensity. Regional analysis using DMRcate identified a region with eight sites in the micro-imprinted locus containing the genes Bladder Cancer Apoptosis Inducing Protein (*BLCAP*) and Neuronatin (*NNAT*), where the encoded proteolipid protein NNAT functions in the control of ion channels during brain development (Table 3, Figure 2D).

### 2.3. EWAS Identifies Differentially Methylated Sites Associated with and Chronic Pain Given Pressure Injury History (Model 2)

**Figure 3:**
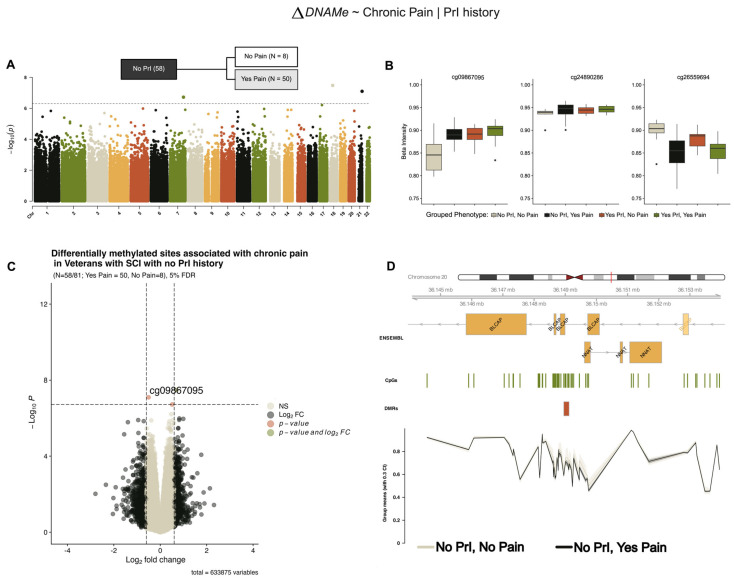
Several differentially methylated sites and a region within the *BLCAP/NNAT* locus are associated with chronic pain in individuals with and no recurrent PrI (model 2). (**A**) Manhattan plot depicting −log10-transformed *p*-values per site in EPIC v1 (hg19) array associated with pain in persons with SCI who have no recurrent PrI history. (**B**) Individual effect plots of median and inter-quartile range (IQR) beta intensity for each significant CpG probe indicate modest effect sizes from individual probes. (**C**) Volcano plot indicates that only one probe meets the significance threshold and log2fold-change threshold for genome-wide significance. (**D**) Differentially methylated region analysis using DMRcate and summary statistics identify one locus associated with chronic pain including BLCAP/NNAT. X-axis corresponds to genomic coordinates from ENSEMBL. The Y-axis of the lowest portion of the panel corresponds to grouped-mean beta intensity values per phenotype sub-group, with 30% confidence intervals. Changes in DNA methylation associated with phenotype groups are denoted as ΔDNAMe. Created in BioRender. Alcorn, M. (2025) https://biorender.com/axzqb8g (accessed on 3 July 2025).

Model 2 (Figure 1B) refined our hypothesis to ask if, given recurrent PrI, are there are DMPs associated with those who experience chronic pain and those that do not. Stratifying our analysis by recurrent PrI, we identified the same three DMPs associated with chronic pain from our model 1 (Figure 3) in individuals with no PrI, which also showed similar effect sizes and log2-fold changes in methylation (Table 4).

### 2.4. EWAS Identifies Differentially Methylated Sites Associated with Chronic Pain in Persons with SCI and Recurrent PrI (Model 2)

**Figure 4:**
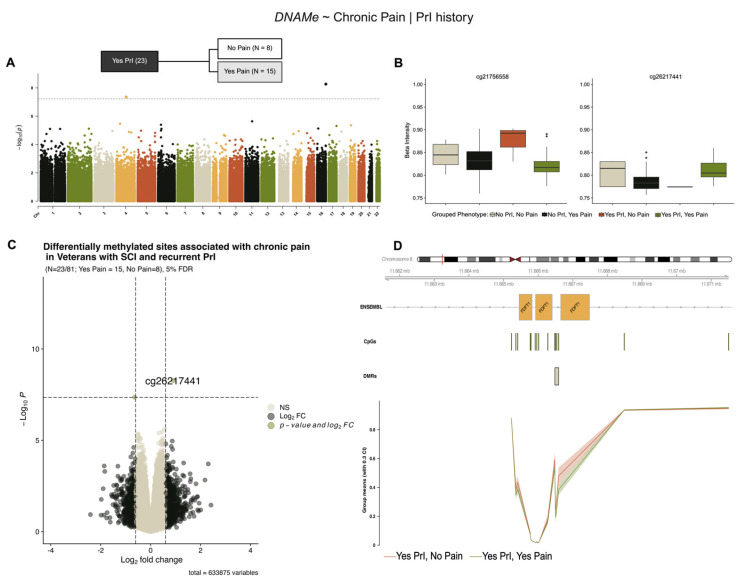
Two differentially methylated sites and a region within *FDFT1* are associated with chronic pain in individuals with SCI and recurrent PrI. (**A**) Manhattan plot depicting −log10-transformed *p*-values per site in EPIC v1 (hg19) array associated with chronic pain in persons with SCI who have recurrent PrI history. (**B**) Individual effect plots of median and inter-quartile range (IQR) beta intensity for each significant CpG probe indicate modest effect sizes from individual probes. (**C**) Volcano plot indicates that only one probe meets the significance threshold and log2fold-change threshold for genome-wide significance. (**D**) Differentially methylated region analysis using DMRcate and summary statistics identify one DMR within the protein coding gene FDFT1 associated with chronic pain. Changes in DNA methylation associated with phenotype groups are denoted as ΔDNAMe. Created in BioRender. Alcorn, M. (2025) https://biorender.com/oxdjrl8 (accessed on 3 July 2025).

Stratification of our cohort by PrI status revealed two novel sites associated with chronic pain in individuals with recurrent PrI (Yes PrI = 23/81, No pain = 8, Yes pain = 15), (Figure 4, Table 5). Figure 4B,C show modest log2-fold changes and methylation alterations, consistent with previous models’ effect sizes. Regional analysis using a sliding window and summary statistics from single-site analysis in *DMRcate* identified five probes associated with the protein-coding gene *Farnesyl-Diphosphate Farnesyltransferase 1* (*FDFT1*), a lynch-pin enzyme involved in cholesterol synthesis (Figure 4D, Table 6).

### 2.5. DMR Analysis in Features Known to Regulate Gene Expression Identifies Several Shared Genes, Promoters and CpG Islands Associated with Recurrent PrI History, Chronic Pain, and Chronic Pain Given Recurrent PrI History

**Figure 5:**
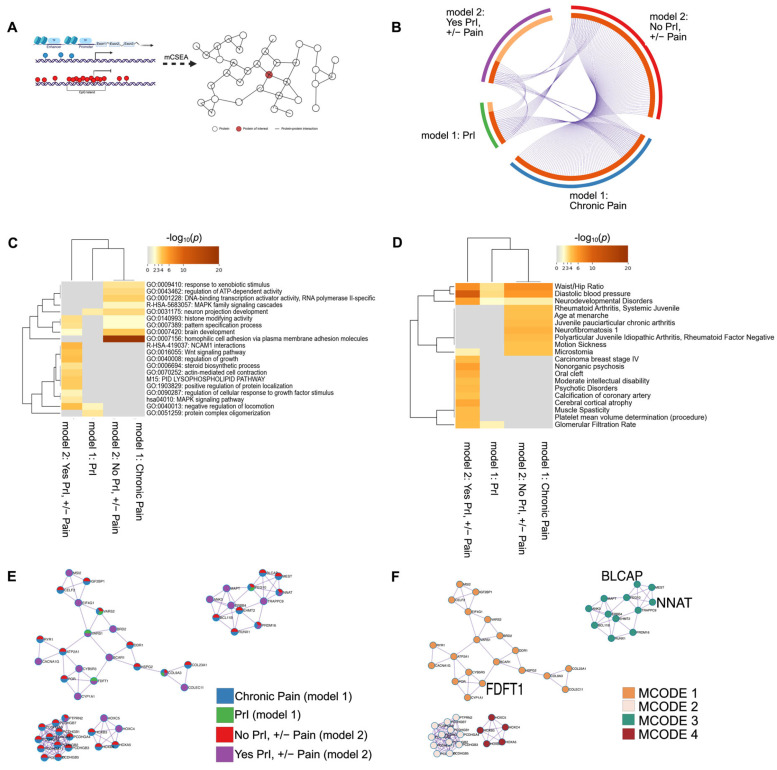
DMR analysis in features known to regulate gene expression identifies several shared genes, promoters and CpG islands associated with recurrent PrI history, chronic pain, and chronic pain given PrI history. *(***A**) mCSEA interrogates probes in pre-defined genomic regions specifically related to changes in gene expression. Genes and genes associated with promoters were used as input for annotation/enrichment analysis and protein–protein interaction network analysis using MetaScape. (**B**) Circos plot shows shared features between phenotype groups: PrI, Pain, and stratified groups by PrI history. (**C**) Heatmap depicting top twenty clusters of gene-ontology terms associated with each phenotype group. *p*-values are unadjusted and log-scaled. (**D**) Heatmap of unadjusted *p*-values for enrichment of genetic features from each phenotype group with genes known with certain pathologies and other conditions. *p*-values are unadjusted and log-scaled. (**E**,**F**) Protein–protein interaction network after being processed by MCODE algorithm using MetaScape. (**E**) Each circle is a pie-graph depicting relative number of genes as input from each phenotype group. (**F**) Each cluster from E depicting associated MCODE term or terms enriched in most densely connected nodes of network (Table 7). Created in MetaScape and BioRender. Alcorn, M. (2025) https://biorender.com/zlhriev (accessed on 3 July 2025).

Many complex, non-malignant traits often depend on input from tens to hundreds of loci with modest effect sizes, making it challenging to identify regions with small but consistent effect sizes that do not reach the genome-wide statistical threshold. To identify these regions as well as those that reached statistical significance (Figure 2, Figure 3 and Figure 4), we used the R package mCSEA (methylated CpGs Set Enrichment Analysis [11]) to search for small but consistent changes in methylation in pre-defined genomic regions, which also reduces the number of overall tests and increases power, an important feature for small cohorts. Using *t*-test statistics calculated by limma to rank individual probes, we used mCSEA to perform burden-testing in pre-defined regions containing at least 30 individual sites. (See Figure 5A and Supplemental Tables S5–S8 for gene-names and enrichment scores per phenotype per model.) Unlike the results from model 1, we were able to identify several genes, promoter regions and CpG islands significantly associated with PrI, chronic pain, and each phenotype sub-group using mCSEA (adjusted *p* < 0.05) (Supplemental Tables S5–S8).

To put the genetic features identified by mCSEA into a larger context, we used MetaScape for annotation and enrichment analysis using genes and genes associated with promoters for each phenotype group: model 1 included PrI, and chronic pain, and stratified phenotype analysis was used in model 2 (No PrI, +/−Pain; Yes PrI, +/−Pain). Thus, we generated a large list of genes and associated promoter regions (Figure 5B, Supplemental Tables S5–S8). We annotated differentially methylated features for function/location using available databases in MetaScape for pathways, molecular function and patterns of gene expression (see Section 4). We also constructed a protein–protein interaction network, which was also generated using CORUM. Terms were included using default MetaScape parameters (*n* > 3 overlapping terms, adjusted *p*-value < 0.01, and minimum enrichment score of 1.5)^3^. Enrichment analysis identified significant overlap of regions, as noted by their gene name or gene associated with the promoter region, between all phenotype groups (Figure 5B).

Enrichment analyses indicate the shared, overlapping network of proteins encoded by genes or genes associated with promoters (Figure 5A, Circos plot) for both sub-group phenotypes and PrI (Figure 5B), which is reflected in the ontology and related traits analyses (Figure 5B,C). Genes associated with both overall chronic pain experience and no PrI history were significantly associated with GO terms for homophilic cell adhesion via plasma membrane adhesion molecules (*p* = 1 × 10^−23^), cell–cell adhesion via plasma-membrane adhesion molecules (*p* = 1 × 10^−20^), calcium ion binding (*p* = 1 × 10^−16^) and cell–cell adhesion (*p* = 1 × 10^−14^), while genes associated with chronic pain in persons with SCI and recurrent PrI were nearly significantly enriched for NCAM1 interactions (*p* = 0.0631). Networks built using DisGeNET found that genes associated with all phenotype groups were also enriched in traits such as diastolic blood pressure (*p* = 7.94 × 10^−6^), waist/hip ratio (*p* = 2.51 × 10^−3^), and neurodevelopmental disorders (*p* = 0.01), while there seems to be a distinct split in enrichment terms between Yes PrI, +/− Pain group and other phenotypes (Figure 5D). The protein–protein interaction network also identified set-specific contributions in the overall network from each phenotype group (Figure 5E,F). Figure 5E,F depicts the network after applying the Molecular Complex Detection (MCODE) to identify densely connected networks within large networks. The count of genes from each phenotype group are shown by pie-graphs, where a unique color represents the relative proportion of genes from each phenotype group within each MCODE designation.

**Table 7:** Final MCODE designation of PPI network constructed using MetaScape.

MCODE	GO #	Description	Log_10_ (*p*-Value)
Final MCODE	GO:0007156	Homophilic cell adhesion via plasma membrane adhesion molecules	−14.6
	GO:0005509	Calcium ion binding|	−14
	GO:0098742	Cell–cell adhesion via plasma-membrane adhesion molecules|	−12.4
Final: MCODE 1	GO:0046209	Nitric oxide metabolic process	−6
	GO:2001057	Reactive nitrogen species metabolic process	−5.9
	WP43	Oxidation by cytochrome P450	−5
Final: MCODE 2	GO:0007156	Homophilic cell adhesion via plasma membrane adhesion molecules	−23.9
	GO:0098742	Cell–cell adhesion via plasma-membrane adhesion molecules	−21.7
	GO:0098609	Cell–cell adhesion	−18.1
Final: MCODE 3	GO:1903829	Positive regulation of protein localization|	−4.5
	GO:0021953	Central nervous system neuron differentiation	−4.2
	GO:0045165	Cell fate commitment|	−3.9
Final: MCODE 4	GO:0048706	Embryonic skeletal system development	−11.8
	GO:0009952	Anterior/posterior pattern specification	−10.8
	GO:0003002	Regionalization|	−9.30

## 3. Discussion

In this work, we sought to model the intersection between two commonly associated secondary health complications in individuals with SCI: PrI and chronic pain. Our main goal was to quantify subjective symptom experiences and measure the epigenetic underpinnings of symptoms to better understand the underlying biology of these complex traits. Single-site analysis identified three probes associated with overall variation in the experience of chronic pain and a single region within the *BLCAP/NNAT* locus. However, stratifying our analysis by PrI revealed that chronic pain in individuals with SCI and recurrent PrI is associated with two novel sites and a region within the protein-coding gene *FDFT1*, while the results from our first model were recapitulated in the group with no PrI history.

Regional analysis focused on a pre-defined set of genes, promoters and CpG islands identified several genes, promoters and CpG islands associated with PrI, chronic pain experience and both sub-groups including *BLCAP/NNAT* and *FDFT1* from model 1 (Supplemental Tables S5–S8). While this second approach is limited to known features, gene ontology and enrichment analysis regions identified by mCSEA suggest that there is a much larger gene regulatory network associated with each phenotype. Interestingly, a multi-ancestry GWAS for pain intensity using a large cohort of over 500,000 Veterans identified variants in protein-coding genes [12]. Genes identified by mCSEA analysis in this study included *GNA12* (PMID: 38429522), *GALNS* (PMID: 38429522), *SDK2*, and *SHB3BP2* (PMID: 38429522) (Supplemental Tables S5–S8). Additionally, mCSEA analysis also identified Interleukin *IL17RD*, a gene encoding a membrane protein belonging to the Interleukin-17 receptor family whose members IL17A and IL17F were identified in a study of chronic widespread pain in mono and dizygotic twins of European descent [13,14]. Taken together, our analysis identifies shared genetic components with previous studies of chronic pain; however, most of our findings may be unique to male individuals with spinal cord injury (Table 1, Supplemental Table S1). Ultimately, these preliminary findings require validation using larger cohorts of individuals with SCI or in vivo validation using an SCI animal model.

The ability to analyze overlapping and interdependent data represents a fantastic opportunity to visualize the complex relationships that exist among SHCs following SCI. Ferguson et al., 2011 introduced “syndromics”, which applies informatics tools to disease models to characterize the full set of mechanistic inter-relationships from multi-scale data (p. 438) [15]. They assert that multivariate approaches are sensitive to associations among multiple outcomes, and such approaches have been widely used to validate genomic patterns as predictors of a particular disease state [15]. Here we used MetaScape to visualize networks of genes or features known to regulate gene expression and found that there are significant overlaps and potentially significant differences amongst all phenotype groups (Figure 5). With increased utilization of machine learning and artificial intelligence models, network analysis is a powerful tool that can help with the development of risk prediction models and improve our understanding of SHCs. Fallah et al., 2024 utilized network models (Gaussian Graphical Model (GGM), Ising Model, and mixed graphical models (MGMs)) to identify key variables linking the SHCs in a Canadian SCI community cohort from 2011 to 2012 [16]. This is the only study of this kind; thus, replication studies using shared bioinformatic tools as well as larger samples would provide an opportunity for validation and clinical translation.

As people with lived SCI continue to live longer and more people sustain SCI later in life, there is a need to better understand the accelerated aging phenotype that SCI represents and how this impacts multimorbidity. DNAm provides a better measure of chronic exposure, making it a strong tool for biomarker analysis. The development of disease in people who sustain SCI at a young age and are aging with SCI compared to those that are aged (>65 years) needs to be understood as there are likely differences. Future work will focus on utilizing several epigenomic clocks that can predict disease or time to event (i.e., multimorbidity), and when combined with machine learning, there is a very favorable opportunity to use these tools in the clinical environment.

Despite the modest sample size, we believe the identification of differentially methylated position and associated gene networks provides valuable preliminary insights into the epigenetic landscape of chronic pain and recurrent PrI in individuals with SCI. These findings lay the groundwork for future studies with larger cohorts that would allow for stratification by gender and ancestry. Because of our strong gender bias in this cohort, only autosomal sites were considered for analysis, and gender was excluded as a covariate.

Our study does not suggest that genomic or epigenomic analysis alone can replace self-reported outcomes or clinical assessments. Rather, we propose that epigenetic biomarkers may serve as complementary tools to enhance our understanding of symptom biology when integrated with functional, psychosocial, and environmental data. We also acknowledge the importance of controlling for general health status and environment exposures, and our findings should be interpreted as exploratory.

## 4. Materials and Methods

### 4.1. Cohort Description

This observational study was conducted across three VA Medical Center sites comprising of 81 Veterans with SCI. Potential participants with open pelvic region PrI at the time of recruitment, presence of a systemic disease or condition known to influence inflammatory biomarkers, such as heart disease or uncontrolled diabetes, and known sensitivity to intravenous (IV) contrast were excluded. At the time of enrollment, a comprehensive profile of clinical and health factors was obtained, together with demographic information relevant to PrI history. See Table 1 and Supplemental Table S1.

### 4.2. Blood Collection

Whole blood was collected using PAXgene Blood RNA tubes (Qiagen, Germantown, MD, USA) and BD Vacutainer K2 EDTA tubes (BD Biosciences, Franklin Lakes, NY, USA). Following collection, tubes were immediately placed in −80 °C storage until RNA and DNA isolation was performed for library preparation.

### 4.3. DNA Isolation and Quality Control for Genome-Wide Analysis

DNA isolation was performed on whole blood samples using the PureLink Genomic Mini Kit (Thermo Fisher Scientific, Waltham, MA, USA). Blood tubes were left to thaw at room temperature for up to 15 min before extracting 200 µL blood for DNA isolation. Following isolation, DNA concentration and quality were measured using the NanoDrop ND-2000 and the Qubit 4 Fluorometer BR DNA assay kit (Thermo Fisher Scientific, Waltham, MA, USA). Samples with A260:280 ratios between 1.8 and 2.1 and concentrations greater than 20 ng/µL were included for analysis. High quality samples were then collected in three separate batches and submitted to Case Western Reserve University Genomics Core (CWRU, Cleveland, OH, 44106, USA) for further analysis. Briefly, DNA was prepped for quantification using the EZ DNA Methylation Kit (Zymo Research Corp, Irvine, CA, USA), and methylation was quantified using either the Illumina Infinium HumanEPIC BeadChip (Illumina Inc., San Diego, CA, USA) or Illumina Infinium HumanMethylationEPICv2 BeadChip (Illumina Inc., CA, USA) using NextSeq 550.

## 5. Data Analysis

All statistical analyses were performed using R version 4.4.0 [17] and Bioconductor 3.19 unless otherwise stated.

### 5.1. Covariate Analysis

To identify specific covariates that may confound analysis, all numeric covariates (age, %IMAT, BMI, weight (lbs.)) were standardized using median and mean-absolute deviation from the median (MADS). The categorical covariates of diabetic and smoking status were recast from the original data to make fewer categories and increase group size. Specifically, smoking status was re-cast to three levels (Never, Former and Current), and diabetic status was recast to (Controlled vs. No or Pre). Pairwise correlation and logistic regression were used to identify any significant covariates associated with recurrent PrI history (Supplemental Tables S2 and S3). All statistical tests were corrected for multiple comparisons using the Benjamini and Hochberg method. We used significantly associated and biologically relevant variables to create five different possible models for genome-wide association analysis (E/GWAS) (Supplemental Table S4).

### 5.2. Methylation Data Processing: Site and Sample QC

The R package sesame [18,19,20] was used to perform site and sample quality control (QC), while limma [21], minfi [22,23,24], DMRcate [25,26,27], and mCSEA were used for differential methylation analysis. Sample and site QC was performed on each batch individually using recommended preprocessing steps per platform (EPICv2 or EPICv1). Briefly, signal intensity from raw idat files from each batch was imported into R version 4.4.0 using the readIDAT() function from the package sesame. Probes were masked (preprocessing code = “QCDPB”) using recommended masks per platform in addition to filtering sites with bead count < 3. Probes were marked for removal if they were non-unique, prone to mapping issues, or in sites where extension or probe-intensity was affected by a SNP. The pOOBAH() function was used to calculate detection *p*-values, and probes with *p* < 0.05 were also masked in parallel with the other QC steps [20]. Proceeding steps removed background noise from beta values, including channel inference, non-linear dye bias correction and normal-exponential deconvolution using out-of-band probes (noob) [28].

Beta values from samples and probes that passed initial QC from EPICv2 batches were then lifted to EPICv1 using the liftOver() function in sesame without imputation. After QC, all beta values from three different batches were merged. Probes were then filtered for autosomal sites and any additional probes that are known for cross-hybridization using the rmCHandSNPs() function in DMRcate, resulting in 633,875 sites for analysis.

Principal component analysis (PCA) using the prcomp() function in R was used to check for any batch effects or outliers using the top two principal components from merged beta values. PCA identified a strong batch effect between the two EPIC platforms. PCA using beta values processed by the removeBatchEffect() function from limma [18] using platform as the batch variable indicates that accounting for platform does indeed remove the batch effect (Supplemental Figures S2 and S3). Thus, platform version (EPICv1 or EPICv2) was used as a covariate in all possible models to consider any residual batch effect (Supplemental Figures S2 and S3).

### 5.3. Epigenome-Wide Association Study (EWAS)

We built five possible models using limma to investigate whether any variation in methylation is associated with the intersection between PrI and chronic pain. The overall model fit and signs of statistical inflation were determined by calculating Lambda GC values of summary statistics for each possible model (Supplemental Table S4), and those with Lambda GC values in the range 0.9 < *n* < 1.10 were considered for analysis and annotation. To account for additional sources of heterogeneity, such as variation in immune-cell types, models also included support vectors calculated using the R-package sva [28]

DMRs were identified using summary statistics from limma using *DMRcate* [26]. DMRs specifically in genes, promoters and CGI were identified using the mCSEA [11] package in R, and probes were ranked by t-values calculated using limma. DMRs identified by DMRcate were considered significant if the region contained more than 5 probes and adjusted *p*-values < 0.05 (5% FDR). DMRs identified by mCSEA were considered significant if region contained more than 30 probes and adjusted *p*-values < 0.05 [11].

### 5.4. Annotation and Enrichment Analyses for Genes Identified by mCSEA

Annotation and enrichment analysis for DMRs (regions containing CpGs included gene body, promoter regions) identified by mCSEA for each phenotype was carried out using MetaScape [29]. Separate lists were combined using meta-analysis, and genes were annotated and tested for enrichment in the following databases: KEGG pathways, GO terms, protein function (Protein Atlas), subcellular location (Protein Atlas), genotype/phenotype/disease (JAX, GOSlim, Orphanet, OMIM, ClinVar, dbGap (NCBI), GWAS Catalog (NHGRI-EBI)), developmental disorders (DDG2P), variations (Ensembl), human phenotype ontology (DisGeNET), disease ontology (GeDiPNet, ChatGPT), and tissue-specific expression (TIGER, Protein Atlas). Terms were considered significant or were used for clustering analysis using default MetaScape parameters (adjusted *p*-value < 0.01, *n* > 3 per enrichment term, enrichment score > 1.5).

## 6. Conclusions

Precision health is a model that considers the individual’s unique genes, environment, and lifestyle to tailor disease prevention and treatment to their individual needs. The goal is to provide the right treatment at the right time, based on the individual’s predicted response or risk of disease. The Precision Medicine Initiative was launched in 2015 and as a result, precision cancer care and cardiovascular care are making significant advances. Unfortunately, precision rehabilitation continues to lag behind, especially in the SCI subspecialty. This is in part due to poor translation of research findings that have clinical utility. While functional biomarkers that measure physical exercise capacity are powerful tools in the precision rehabilitation toolbox, the need for clinical biomarkers continues to be a challenge. The potential of epigenetic markers is currently preliminary and exploratory but lays a foundation for complementary tools that may, in time, enhance the precision of symptom science when integrated with existing clinical frameworks.

## Figures and Tables

**Table 1:** Study participant demographics and clinical information for EWAS analysis of participants from initial visit (N = 81).

		No PrI History, No Chronic Pain (N = 8)	No PrI History, Yes Chronic Pain (N = 50)	Recurrent PrI History, No Chronic Pain (N = 8)	Recurrent PrI History, Yes Chronic Pain (N = 15)
Collection Site (VAMC)	Cleveland	4	30	6	15
	Bronx	2	10	2	1
	Minneapolis	2	10	-	1
Age	Median (years), (IQR)	43.5 (23.875)	48.50 (40.750)	25.25 (40.375)	29.50 (26.000)
Gender	M	8	49	8	15
	F	-	1	-	2
Ancestry (self-reported)	European	7	35	6	14
	African American or LatinX	1	15	2	3
IMAT	Median%, IQR	26 (38)	9.5 (16.5)	15.5 (19.5)	23 (30.5)
AIS	Incomplete	6	43	4	7
	Complete	1	7	4	10
	NA	1	-	-	-
Level of Injury	Above T6	6	39	5	13
	Below T6	2	11	3	4
Length of Injury	Median (years), IQR	43.0 (32.875)	44.5 (38.375)	38.0 (43.250)	41.00 (80.00)

**Table 2:** Top three differentially methylated sites associated with chronic pain (model 1).

Probe Name	cg09867095	cg26559694	cg24890286
Chromosome	chr18	chr7	chr21
Position	28470269	139317303	47739342
Strand	-	-	+
*p* Value	3.32 × 10^−8^	7.98 × 10^−8^	1.91 × 10^−7^
Adjusted *p* value	0.021	0.025	0.04
Log_2_ Fold Change (95% CI)	0.698 (0.4750, 0.9218)	−0.502 (−0.6670, −0.3360)	0.513 (0.3370, 0.6902)
Island Name			chr21:47742779–47743269
Relation to Island	Open Sea	Open Sea	N. Shelf
UCSC RefGene Name		*HIPK2*	*C21orf58*
UCSC RefGene Group		Body	5′UTR;TSS1500;Body

**Table 3:** Regional analysis using genome-wide sites for entire cohort identifies one locus associated with chronic pain (model 1).

Chromosome	chr20
Start (bp)	36148954
End (bp)	36149121
Width (bp)	168
Number of CpGs within DMR	8
Minimum FDR of the smoothed estimate.	1.063 × 10^−27^
Harmonic mean of the individual CpG *p*-values	0.00243
Maximum differential/coefficient within the DMR	−0.095
Mean differential/coefficient within the DMR	−0.065
Overlapping Genes	*BLCAP*

**Table 4:** Top differentially methylated sites associated with chronic pain in study cohort without recurrent PrI.

Probe Name	cg09867095	cg24890286	cg26559694
Chromosome	chr18	chr7	chr21
Position (bp)	28470269	139317303	47739342
Strand	-	-	+
*p* Value	3.32 × 10^−8^	1.91 × 10^−7^	7.98 × 10^−8^
Adjusted *p* Value	0.021	0.04	0.025
Log_2_ Fold Change (95% CI)	0.698 (0.475, 0.921)	0.513 (0.337, 0.69)	−0.502 (−0.668, −0.335)
Island Name			chr21:47742779–47743269
Relation to Island	Open Sea	Open Sea	N. Shelf
UCSC RefGene: Name		*HIPK2*	*C21orf58*
UCSC RefGene: Group		Body	5′UTR;TSS1500;Body

**Table 5:** Top differentially methylated sites associated with chronic pain in study cohort with recurrent pressure injuries using stratified model 2.

Probe Name	cg21756558	cg26217441
Chromosome	chr4	chr16
Position (bp)	101502764	89643414
Strand	-	-
*p* Value	4.41 × 10^−8^	5.43 × 10^−9^
Adjusted *p* Value	0.0140	0.00344
Log_2_ Fold Change (95% CI)	−0.644 (−0.852, −0.436)	0.901 (0.632, 1.170)
Island Name	na	
Relation to Island	Open Sea	Island
UCSC RefGene: Name	na	*CPNE7*
UCSC RefGene: Group	na	Body
Gencode v12: Name	*EMCN*	na
Gencode v12: Group	3′UTR	na

**Table 6:** Top differentially methylated regions associated with chronic pain in study cohort given propensity for PrI (model 2).

Phenotype Group:	Yes PrI, +/−Pain	No PrI, +/−Pain
Chromosome	chr8	chr20
DMR: Start (bp)	11666485	36148954
DMR: End (bp)	11666594	36149121
Width (bp)	110	168
Number of CpGs within DMR	5	8
Minimum FDR of the smoothed estimate	4.15 × 10^−11^	1.063 × 10^−27^
Harmonic mean of the individual CpG *p*-values	0.000240	0.0024
Maximum differential/coefficient within the DMR	−0.13	−0.095
Mean differential/coefficient within the DMR	−0.093	−0.066
Overlapping Genes	*FDFT1*	*BLCAP*

## Data Availability

Data available on request due to privacy restrictions.

## Supplementary Materials

The following supporting information can be downloaded at https://www.mdpi.com/article/10.3390/epigenomes9030026/s1. Figure S1: Pair-wise correlation analysis identifies several significant covariates with recurrent pressure injury status (PrI) history in persons with SCI; Figure S2: PC analysis identifies strong batch effect associated with sequencing platform used per batch; Figure S3: PC analysis of beta values after removing batch effect for sample platform shows reduced batch effect. Table S1: Demographic information for 81 Veterans with SCI/D stratified by recurrent PrI and chronic pain (Y/N) at time of enrollment. Table S2: Pair-wise correlation analysis using Kendall’s tau and false discovery rate to compare for all pair-wise correlations. Variables in black box were used in a potential model for EWAS; Table S3: Logistic regression of PrI phenotype. Table S4: Possible models for EWAS and associated Lambda GC statistics for each phenotype of interest; Table S5: mCSEA enrichment statistics for genes, promoters and CpG Islands associated with overall pain experience (model 1). Genes with *n* > 30 CpGs in the pre-defined region and adjusted *p*-values < 0.05 (5% FDR); Table S6: mCSEA enrichment statistics for genes, promoters and CpG Islands associated with recurrent PrI (model 1). Genes with n > 30 CpGs in the pre-defined region and adjusted *p*-values < 0.05 (5% FDR); Table S7: mCSEA enrichment statistics for genes, promoters and CpG Islands associated with pain in participants with no recurrent PrI (model 2). Features with *n* > 30 CpGs in the pre-defined region and adjusted *p*-values < 0.05 (5% FDR); Table S8: mCSEA enrichment statistics for genes, promoters and CpG Islands associated with chronic pain in persons with recurrent PrI. Genes with *n* > 30 CpGs in the pre-defined region and adjusted *p*-values < 0.05 (5% FDR); Table S9: Data dictionary for variables included in pair-wise correlation analysis (ST2) and logistic regression (ST3)
